# Enhanced dynamic functional connectivity (whole-brain chronnectome) in chess experts

**DOI:** 10.1038/s41598-020-63984-8

**Published:** 2020-04-27

**Authors:** Enrico Premi, Stefano Gazzina, Matteo Diano, Andrea Girelli, Vince D. Calhoun, Armin Iraji, Qiyong Gong, Kaiming Li, Franco Cauda, Roberto Gasparotti, Alessandro Padovani, Barbara Borroni, Mauro Magoni

**Affiliations:** 1 Stroke Unit, Azienda Socio Sanitaria Territoriale Spedali Civili, Spedali Civili Hospital, Brescia, Italy; 2 Neurophysiology Unit, Azienda Socio Sanitaria Territoriale Spedali Civili, Spedali Civili Hospital, Brescia, Italy; 3 Department of Psychology, University of Turin, Turin, Italy; 4 TakeAppWay, Manerbio, Italy; 5 Tri-institutional center for Translational Research in Neuroimaging and Data Science (TReNDS), Georgia State University, Georgia Institute of Technology, and Emory University, Atlanta, Georgia USA; 6 Huaxi MR Research Center, Section of Neuroradiology, Department of Radiology, West China Hospital of Sichuan University, Sichuan, China; 7 GCS fMRI, Koelliker Hospital and University of Turin, Turin, Italy; 8 Focus Lab, Department of Psychology, University of Turin, Turin, Italy; 9 Neuroradiology Unit, Department of Medical-Surgical Specialties, Radiological Sciences and Public Health, University of Brescia, Brescia, Italy; 10 Centre for Neurodegenerative Disorders, Neurology Unit, Department of Clinical and Experimental Sciences, University of Brescia, Brescia, Italy

**Keywords:** Computational models, Network models

## Abstract

Multidisciplinary approaches have demonstrated that the brain is potentially modulated by the long-term acquisition and practice of specific skills. Chess playing can be considered a paradigm for shaping brain function, with complex interactions among brain networks possibly enhancing cognitive processing. Dynamic network analysis based on resting-state magnetic resonance imaging (rs-fMRI) can be useful to explore the effect of chess playing on whole-brain fluidity/dynamism (the chronnectome). Dynamic connectivity parameters of 18 professional chess players and 20 beginner chess players were evaluated applying spatial independent component analysis (sICA), sliding-time window correlation, and meta-state approaches to rs-fMRI data. Four indexes of meta-state dynamic fluidity were studied: *i)* the number of distinct meta-states a subject pass through, *ii)* the number of switches from one meta-state to another, *iii)* the span of the realized meta-states (the largest distance between two meta-states that subjects occupied), and *iv)* the total distance travelled in the state space. Professional chess players exhibited an increased dynamic fluidity, expressed as a higher number of occupied meta-states (meta-state numbers, 75.8 ± 7.9 vs 68.8 ± 12.0, p = 0.043 FDR-corrected) and changes from one meta-state to another (meta-state changes, 77.1 ± 7.3 vs 71.2 ± 11.0, p = 0.043 FDR-corrected) than beginner chess players. Furthermore, professional chess players exhibited an increased dynamic range, with increased traveling between successive meta-states (meta-state total distance, 131.7 ± 17.8 vs 108.7 ± 19.7, p = 0.0004 FDR-corrected). Chess playing may induce changes in brain activity through the modulation of the chronnectome. Future studies are warranted to evaluate if these potential effects lead to enhanced cognitive processing and if “gaming” might be used as a treatment in clinical practice.

## Introduction

Neuroplasticity has always been considered as one of the most intriguing characteristics of the human brain^1–4^. In the last years, multidisciplinary research efforts have progressively demonstrated that the brain is potentially modulable by the long-term acquisition and practice of specific skills^5,6^. From this point of view, several studies have clearly reported how chess playing can be considered a paradigm that may induce long term changes in the brain. Indeed, advanced chess players can simulate/imagine the best next moves starting from a reservoir of chess patterns (chunks)^7^ through the involvement of high-level cognitive functions such as planning future actions, visuo-spatial perception, working memory, problem solving, judgment and decision making, and selection of previously acquired schemes^7–12^.

Continuous practice can boost these specific cognitive processes, with consequent enhancement of neuroplasticity mechanisms^13,14^. In particular, neuroimaging studies have shed light on how the brain is engaged during chess playing^7,15,16^ and how the brain undergoes long term reshaping due to practice^8–10,17^. Interestingly, chess players show complex interactions among brain networks with *i)* greater deactivation of the default mode network (DMN) and enhanced striatal-DMN integration during problem-solving^8,9^, *ii*) increased hub functional connectivity between the posterior fusiform gyrus and visuospatial attention and motor networks^17,18^, and *iii*) a more efficient whole-brain organization (increased small-world topology)^10^ as compared to healthy volunteers not engaged in chess playing.

However, functional connectivity approaches usually rely on the conceptual framework that the functional coupling among brain regions is a static feature, with no change over short periods of time^19–21^. In the last few years, this paradigm has been shown to be simplistic, as methodological approaches able to study the human brain as an interacting dynamic system have been developed (dynamic functional network connectivity (dFNC), the chronnectome)^22–28^. Cross-network correlations on successive sliding windows from the original scan-length network time-courses have revealed reproducible reccurring patterns of brain functional connectivity (time-varying connectivity)^24,29,30^. Recently, to incorporate key features of dynamic functional connectivity, the meta-state approach has been proposed^31^: for each subject, at a given point in time, the weighted probability to be in more than one state (distribution of probability of meta-state)^31^ can be defined. Briefly, with this more flexible approach multiple states might be represented to varying degrees at the same point in time, exhibiting lesser distortion in the features under investigation since contributions of all overlapping states are considered^32^, also providing a much more condensed summary measure of dynamic functional connectivity. In this view, objective measures of meta-state dynamic fluidity may be computed, such as the number of meta-states a subject passes through or the overall distance travelled by each subject through the state space. This highly reproducible approach^31,33^ has been already applied to schizophrenia^31^ and to neurodegenerative disorders^34^ and has helped in further understanding the neural basis of brain functioning^22,35–38^.

These premises set the stage for the present work, where we used the meta-state dynamic connectivity approach to explore differences between professional and beginner chess players, considering whole-brain fluidity/dynamism (the chronnectome).

## Methods

### Subjects

Data were derived from a public dataset on chess players^39^ encompassing professional chess players with a regular training (training time: 4.17 ± 1.72 h/day) and a control group of sex- and age-matched beginner chess players, who knew rules and simple strategies of Chinese chess. Dataset access was made from the “B1000 Functional Connectomes Project” (https://www.nitrc.org/projects/fcon_1000/). Subjects were recruited from the First National Intelligence Games held in Chengdu, China. All participants were right-handed and had no history of psychiatric or neurological disorders. No differences on observation skills or clear-thinking ability were found between these two groups, as already published^39^. Written informed consent was obtained from each subject and approval was obtained through the local Institutional Review Board of the West China Hospital of Sichuan University. Studies performed on this dataset are in accordance with relevant guidelines and regulations. Detailed information on this dataset has been reported in Li and colleagues’ study^39^.

### Magnetic resonance Imaging (MRI) acquisition and preprocessing

MRI data were acquired on a 3T Siemens Trio system (Erlangen, German) at the MR Research Center of West China Hospital of Sichuan University, Chengdu, China. All MRI scans were performed when subjects were relaxed with their eyes open and fixated on a cross-hair centered on the screen. A T2-weighted gradient- echo echo-planar pulse sequence was used to obtain functional MRI (fMRI) images. A total of 205 whole brain echo-planar pulse sequence volumes were acquired using the following parameters: TR = 2000 ms, TE = 30 ms, flip angle = 90°, axial slice thickness = 5 mm, with no gap, slice number = 30, voxel size = 3.75 × 3.75 × 5 mm^3^. Functional preprocessing was carried out as previously published (for each subject, the first 5 volumes of the fMRI series were removed to account for magnetization equilibration; the remaining 200 volumes (total acquisition time: 6 minutes and 40 seconds) underwent slice-timing correction and were realigned to the first volume)^34^, using the toolbox for data preprocessing and analysis for brain imaging (DPABI, http://rfmri.org/dpabi)^40^ based on the Statistical Parametric Mapping (SPM12) software. Taking into account the significant impact of head motion on resting-state fMRI^41,42^, we considered absolute (mean translation and mean rotation) and relative (framewise displacement, FD-P)^42^ motion parameters. We consequently applied four levels of correction based on motion parameters: *i*) the absolute motion cut-off: any subject who had an absolute maximum displacement in any direction larger than 2.5 mm, or a maximum rotation (x,y,z) larger than 2.5°, was excluded from the study; *ii*) the relative motion cut-off: the framewise displacement of head position index (FD-P) (calculated as the sum of the absolute values of the 6 translational and rotational realignment parameters)^42^ with a cut-off of mean FD ≤ 0.2 mm, excluding subjects beyond this limit; *iii*) 12-motion parameters (6 original motion parameters and the 6 first-order derivatives) were applied on networks time-courses: the single time-courses were detrended (to remove baseline drifts from the scanners and/or physiological pulsations) and orthogonalized with respect to 12-motion parameters; and *iv*) the FD-P index previously calculated^42^ for each subject was included as nuisance variable in the final statistical analysis. Data were subsequently spatially normalized to the EPI unified segmentation template in Montreal Neurological Institute coordinates derived from SPM12 software and resampled to 3 × 3 × 3 mm^3^ cubic voxels. Spatial smoothing with an isotropic Gaussian kernel with full width at half-maximum (FWHM) 10 mm was applied.

### Functional network decomposition

As previously reported, the functional imaging data were preprocessed using GIFT (GIFT toolbox, http://trendscenter.org/software/gift)^43^ and a spatially constrained ICA algorithm^44^. Spatially constrained ICA was used to compute intrinsic connectivity networks (ICNs) that corresponded to those from a previous analysis on a very large dataset of healthy subjects for test-retest reliability (37 ICNs derived from 7500 healthy subjects as spatial references, see Supplementary Fig. 1 for the visualization of the spatial maps of the ICNs used)^34,45–47^. Subject-specific spatial patterns and time-courses were calculated and then converted to Z-scores. As already described above, the single time-courses were detrended, orthogonalized with respect to 12-motion parameters, despiked (replacement of outlier time points with 3rd order spline fitting to clean neighboring points) and filtered using a 5th order Butterworth filter (0.01–0.15 Hz)^45^.

### Meta-state computation

Dynamic functional network connectivity (dFNC) was obtained using the dynamic FNC toolbox implemented in GIFT^48^. dFNC was calculated using a sliding-window approach to estimate functional connectivity between ICNs for each segment. Segments were defined with a tapered window convolving a rectangle (width = 30, TRs = 60 s) with a Gaussian (σ = 3 TRs) and slides in steps of 1 TR. A LASSO approach with L1 regularization (100 repetitions) was used to compute the covariance between the independent component (IC) time-courses. For each subject, the optimal regularization parameter (λ) obtained using cross-validation was defined, as previously published^29,49^. To decompose dFNC into connectivity patterns (CPs, meta-states), the sICA approach was applied, considering a number of CPs equal to 5, in line with previous work on meta-states in dynamic brain connectivity, to have a good balance to take into account complex linearly additive effects and to retain richly featured basis pattern^31,33,34^. As previously described, the time-courses were discretized (to work over a more tractable space) into 8 bins (positive and negative quartiles) and each timepoint was ended into a meta-state^33^. The time-courses for sICA CPs were derived from the regression of each subject’s dFNC information at each time window on the group of sICA CPs.

In line with meta-states calculation, each subject will have a weighted probability to be in more than one state, and this time-varying distribution of probability represents the methodological underpinning to obtain effective measures of meta-state dynamic fluidity.

For this purpose, four indexes of connectivity dynamism were herein explored: *i)* the number of distinct meta-states the subjects occupied during their scans (meta-state number); *ii)* the number of times that subjects switched from one meta-state to another (meta-state changes); *iii)* the largest distance between two meta-states that subjects occupied (meta-state span); and *iv)* the overall distance travelled by each subject through the state space (the sum of the L1 distances between successive meta-states, i.e., meta-state total distance)^33^. Moreover, to further test the robustness of the approach on the present data we also considered different model dimensionality (using a number of CPs from 4 to 8): for each number of CPs, the statistical comparisons between professional chess players and beginner chess players were performed.

### Statistical analysis

Comparisons of demographic and clinical characteristics among groups (professional chess players vs. beginner chess players) were assessed by Mann-Whitney U test for continuous variables and χ^2^ test for categorical variables. A general linear model (GLM) considering gender and FD-P as nuisance variables was applied to study dFNC in the two groups. Partial Pearson’s correlation analysis was used to assess the relationship between the meta-state measures (meta-state number, meta-state changes, meta-state span and meta-state total distance) and the total amount of time spent by professional chess players for training, corrected for gender and FD. Statistical analyses were performed by using SPSS software (IBM SPSS Statistics 22.0, Chicago, USA) and statistical significance was set at p < 0.05, considering correction for multiple comparisons (Benjamini-Hochberg False Discovery Rate-FDR correction) for indexes of connectivity dynamism^50^.

## Results

Considering the original group^39^ of 29 professional chess players and 29 beginner chess players, 20 subjects were excluded for technical reasons, namely 2 beginner chess players for different MRI protocol (number of slices), 1 professional chess player for excessive absolute motion, 7 beginner chess players and 10 professional chess players for excessive relative motion (FD > 0.2 mm). Thus, 38 subjects (18 professional chess players and 20 beginner chess players) were considered (Table 1).

**Table 1:** Demographic characteristics and rs-fMRI motion parameters.

Variable	professional chess players	beginner chess players	P-value
Number of subjects	18	20	—
Age, years	27.50 ± 8.20	25.40 ± 6.50	0.55*
Gender, F% (n)	27.80 (5)	65.00 (13)	**0.03**^
Chess training, hours	4.06 ± 1.65	—	—
Education, years	13.28 ± 2.53	14.20 ± 2.46	0.12*
FD-P (Power)	0.14 ± 0.04	0.16 ± 0.04	0.25*
FD-P (Power) >0.5, n	4.3 ± 5.0	2.5 ± 3.0	0.20*
FD-P (Power) >0.5, %	0.021 ± 0.025	0.013 ± 0.015	0.19*

FD = framewise displacement; DVARS = D for the temporal derivative of time-courses, VARS referring to RMS, root mean squared head position change; F = female. Results are expressed by mean  ±  standard deviation, otherwise specified. *Mann-Whitney U test; ^Chi-square test.

**Figure 1:**
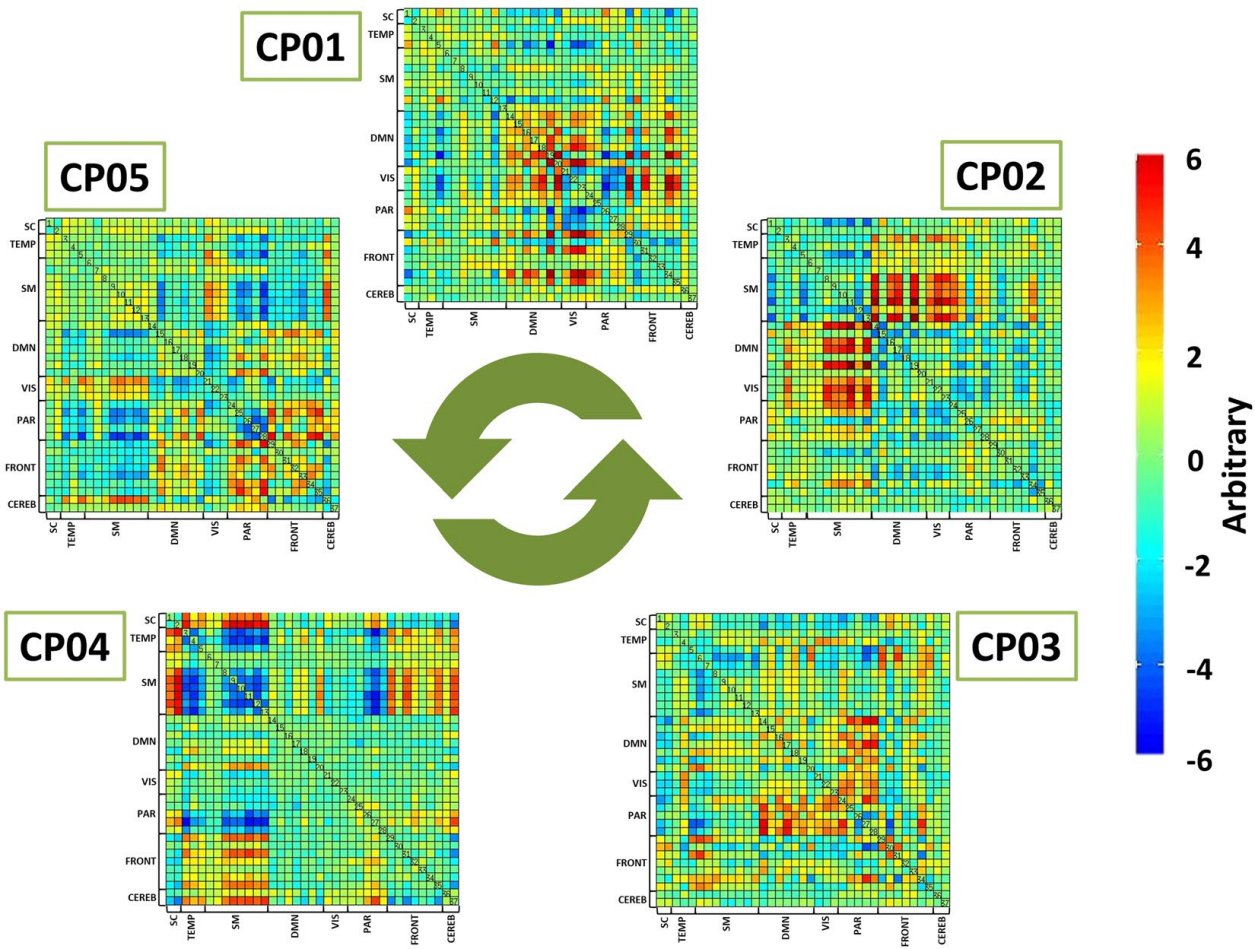
The five connectivity patterns (CPs) resulting from the dynamic Functional Network Connectivity (dFNC) analysis. The five correlation matrices are reported, in which each square represents one of the 37 considered network components. The colors of each CP represent the direction and the strength of the relationship between each dFNC pair and time-course of the CP (red: positive correlation of the time-course; blue: negative correlation of the time-course).

**Figure 2:**
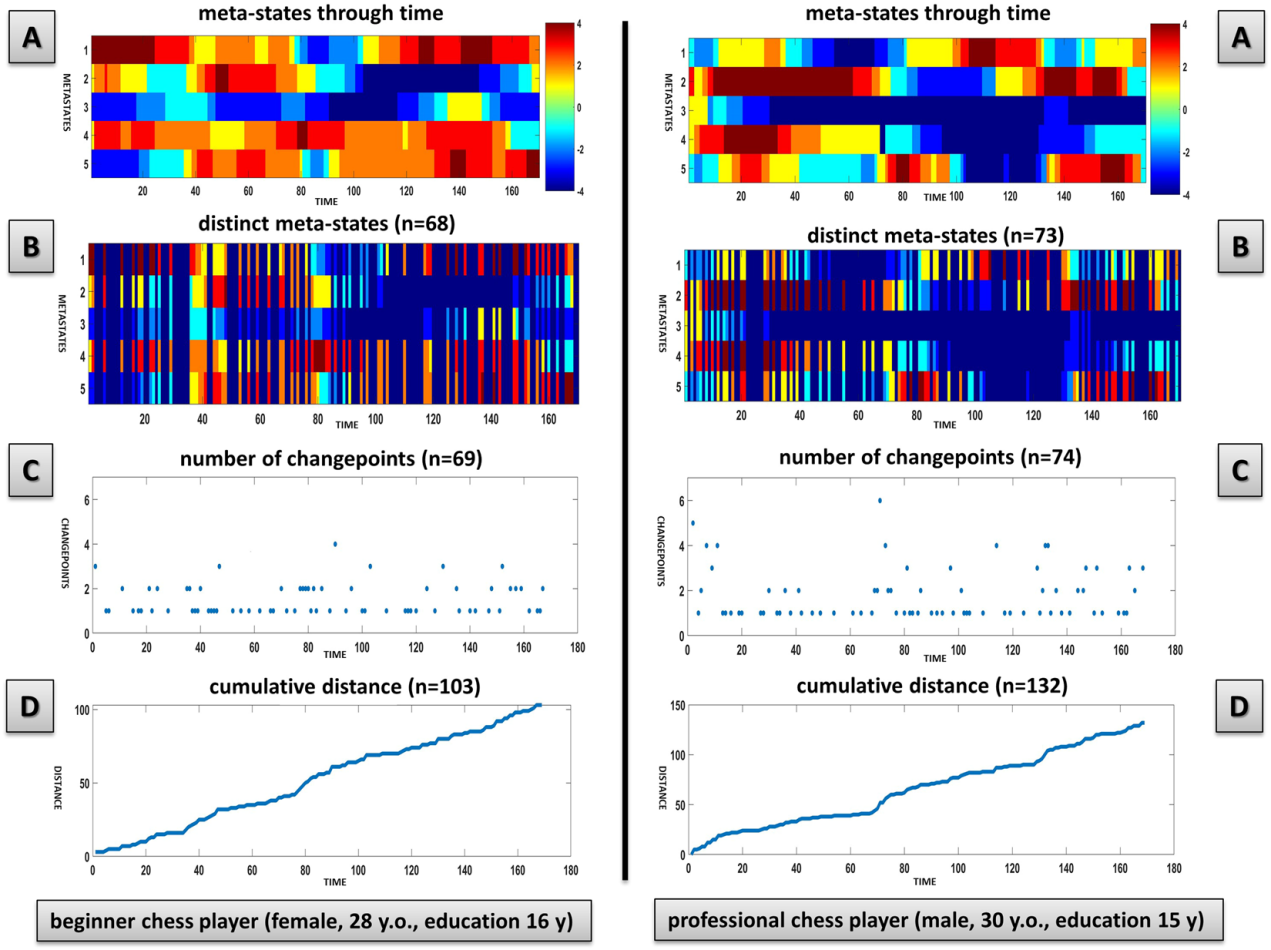
Meta-state dynamics through time, meta-state numbers, meta-state changes, and meta-state total distance in a representative beginner chess player and a representative professional chess player. Meta-state dynamics through time (panel A), meta-state numbers (panel B), meta-state change points (panel C), and meta-state total distance (panel D) in a representative beginner chess player (left column) and in a representative professional chess player (right column). The colorbar represents the strength of probability to be in each meta-state. For panel A and B Y-axis represents the five connectivity patterns (CPs), from 1 to 5 and X-axis represented the time-indexed meta-states (seconds, after time-course discretization in quartiles). For panel C Y-axis represents the distance of each changepoint, whereas the sum of all the blue dots represents the cumulative number of changepoints for a given subject. Finally, for panel D the total cumulative distance traveled (summed L1 distance between successive meta-states) in the state space is reported on Y-axis. y = years; y.o.= years old.

We considered five connectivity patterns (CPs) of dFNC, which are reported in Fig. 1. dFNC was expressed as a weighted sum of the discretized five-dimensional CPs, considering that 8^5^ = 32,768 distinct five-dimensional meta-states in our signed quartile discretization were present, with a mean number of occupied meta-states in the overall group of 72.1 ± 10.8 (0.22% of the total). Professional chess players showed greater dynamic fluidity, as they occupied a higher number of meta-states (i.e., meta-state numbers, p = 0.043 FDR-corrected) and changed from one meta-state to another more often (i.e., meta-state changes, p = 0.043 FDR-corrected) than beginner chess players (see Table 2 and Supplementary Fig. 2). Furthermore, professional chess players operated over an increased dynamic range with increased meta-state total distance (p = 0.0004 FDR-corrected), as they travelled more overall distance, between successive meta-states, through the state space than beginner chess players (see Table 2 and Supplementary Fig. 2). We did not find a statistically significant difference in meta-state span between groups. Considering different numbers of CPs (ranging from 4 to 8) the statistical differences between professional and beginner chess players were quite stable, in particular for meta-state total distance (see Supplementary Table 1 for details). In Fig. 2, meta-state dynamics through time, meta-state numbers, meta-state change points, and meta-state total distance in a representative beginner chess player (on the left) and in a representative professional chess player (on the right) were reported. The representative professional chess player showed a greater brain dynamism, as compared to the beginner chess player (panel A), as suggested by the more complex pattern in the former subject, with a higher number of realized meta-states (panel B), meta-state changes (panel C), and greater travelled overall distance (panel D).

**Table 2:** Meta-state measures in the studied groups.

Variable	professional chess players (n = 18)	beginner chess players (n = 20)	p
Number of distinct meta-states	75.8 ± 7.9	68.8 ± 12.0	0.043*
Number of meta-state changes	77.1 ± 7.3	71.2 ± 11.0	0.043*
Meta-state span	25.5 ± 2.4	23.3 ± 3.8	0.094*
Meta-state total distance	131.7 ± 17.8	108.7 ± 19.7	0.0004*

^*^General Linear Model considering gender and FD-P as covariates of no interest (chess players vs chess novices), FDR-corrected for multiple comparisons. Results are expressed by mean ± standard deviation.

No significant correlation between time spent by professional chess players for daily training and meta-state measures (partial Pearson’s correlation analysis corrected for gender and FD-P: meta-state number: r = 0.150, p = 0.580; meta-state changes: r = 0.195, p = 0.469; meta-state span: r = 0.295, p = 0.268; meta-state total distance: r = 0.055, p = 0.839) was found.

In the whole group no significant correlation between education and meta state measures (partial Pearson’s correlation analysis corrected for gender, chess group and FD-P; meta-state number: r = 0.028, p = 0.872; meta-state changes: r = 0.067, p = 0.661; meta-state span: r = −0.023, p = 0.895; meta-state total distance: r = 0.195, p = 0.261)) was found.

## Discussion

Different board games have been taken into consideration as potential tools to reinforce/strength cognitive abilities in either healthy or diseased brain^51–54^. Among all, the game of chess has been assumed as a paradigm in the field, especially because of its high diffusion worldwide with a significant number of professional players^55^. Several brain hotspots have been demonstrated to be selectively strengthen throughout practice of chess playing, mainly in the basal ganglia, the fusiform gyrus, the default mode network and the attention network^8,9,17^. This reflects the specific abilities employed by professional players/grand masters, based on the simulation and planning of the next best move using previously acquired chess pattern schemes^56^.

Our study tries to move forward, with the idea that regardless of the specific involved brain hubs/networks^8,9,17^, chess playing can potentially modulate whole-brain activity, shaping the spontaneous and time-varying fluctuation of brain signal^22,35,37,57^. In fact, compared to beginners, professional chess players demonstrated enhanced global dynamic fluidity (with a higher number of occupied meta-states, an increased number of changes from one meta-state to another), operating over an extended dynamic range (increased meta-state total distance travelled between successive meta-states). This is in line with a previous study demonstrating an increased small-world topology in chess players, reflecting an optimized cost efficiency of information processing as well as an optimal global network organization as a result of cognitive training^10^. It is interesting to note that static and dynamic brain connectivity are intimately linked, the fine-grained and multi-level organization of the brain based on the spontaneous fluctuation of brain signal^21,58,59^. Considering the theoretic framework of meta-states, these findings suggest that chess playing does not merely modulate brain areas strictly involved in chess practice (such as visuo-attentional network or frontal executive functions) but is able to shape whole brain functioning at different spatial and temporal scales (as suggested in particular by meta-state total distance modulation). Plasticity induced by practice produces multiple structural change in the brain such as myelin reorganization and formation, dendritic branching and synaptogenesis^60^ which boost the effectivity in the neural communication. This process may maximize neuronal tuning, resulting in magnifying transmission and information capacity to a critical optimal point^61^. From this point of view, dynamic functional connectivity and its related measures could be more effective in detecting this functional reorganization.

Furthermore, the impact on brain fluidity and dynamism (as measured by meta-state indexes) is not directly related to the total amount of time spent by chess players in training as well as it is not related to education. Even if the lack of a significant correlation could be related to the small sample size, it might be hypothesized an add-on modulating effect of chess playing, along with the well-established role of education, on cognitive reserve mechanisms^62–64^. Thus, the increased global brain dynamic fluidity observed in professional chess players could provide a direct evidence of neuroplasticity mechanisms related to long-term skill acquisition, potentially representing cognitive reserve enhancement^65–70^. In this view, chess playing may be considered a proxy measure of cognitive reserve along with education, occupation attainment or leisure activities^71–73^ and in some way may protect the brain from physiological or pathological aging^74^.

We acknowledge that this study entails some limits. First, we considered a small number of subjects and further studies are warranted to validate the present findings, considering different level of confounders (age, educational level, etc.). Moreover, impact of head movement on dynamic functional connectivity needs to be further addressed, although in this study we applied strict head motion parameters to exclude this possible bias.

In conclusion, this study demonstrated that chess playing may ameliorate whole brain functioning and increase whole brain dynamic fluidity. Further studies (collecting data with more time-points as well as a higher spatial resolution and applying different dynamic connectivity methodological approaches^75,76^, in different populations and in other board games) may further show how the brain can react and modify itself through the continuous practice of high-level activities with long-term skill acquisition^77^.

## Supplementary information

Supplementary Materials.
