# Supplementary material for: Enhanced dynamic functional connectivity (whole-brain chronnectome) in chess experts
Source: Sci Rep. 2020 Apr 27;10:7051. doi: 10.1038/s41598-020-63984-8 (PMC7184623; doi:10.1038/s41598-020-63984-8)

Enhanced dynamic functional connectivity (whole-brain chronnectome) in chess experts.

Enrico Premi[1*], Stefano Gazzina[2], Matteo Diano[3], Andrea Girelli[4], Vince D. Calhoun[5], Armin Iraji[5], Qiyong Gong[6], Kaiming Li[6], Franco Cauda[7,8], Roberto Gasparotti[9], Alessandro Padovani[10], Barbara Borroni[10], Mauro Magoni[1]

[1] Stroke Unit, Azienda Socio Sanitaria Territoriale Spedali Civili, Spedali Civili Hospital, Brescia, Italy

[2] Neurophysiology Unit, Azienda Socio Sanitaria Territoriale Spedali Civili, Spedali Civili Hospital, Brescia, Italy

[3] Department of Psychology, University of Turin, Turin, Italy

[4] TakeAppWay, Manerbio, Italy

[5] Tri-institutional center for Translational Research in Neuroimaging and Data Science (TReNDS), Georgia State University, Georgia Institute of Technology, and Emory University, Atlanta, Georgia, USA

[6] Huaxi MR Research Center, Section of Neuroradiology, Department of Radiology, West China Hospital of Sichuan University, China

[7] GCS fMRI, Koelliker Hospital and University of Turin, Turin, Italy

[8] Focus Lab, Department of Psychology, University of Turin, Turin, Italy

[9] Neuroradiology Unit, Department of Medical-Surgical Specialties, Radiological Sciences and Public Health, University of Brescia, Italy

[10] Centre for Neurodegenerative Disorders, Neurology Unit, Department of Clinical and Experimental Sciences, University of Brescia, Brescia, Italy

Supplementary Table 1. Meta-state measures' statistical comparisons (professional chess players vs beginner chess players) considering different dimensionality (using sICA approach with a number of CPs ranging from 4 to 8).

	meta-states, number	meta-states, changes	meta-states, span	meta-states, total distance
CPs=4	0.05	0.05	0.22	0.05
CPs=5	0.043	0.043	0.094	0.0004
CPs=6	0.06	0.06	0.61	0.0003
CPs=7	0.06	0.06	0.85	0.06
CPs=8	0.07	0.06	0.88	0.001

*General Linear Model considering gender and FD-P as covariates of no interest, FDR-corrected for multiple comparisons were reported (professional chess players vs beginner chess players). Statistically significant comparisons (p<0.05 FDR-corrected) were shown in grey. sICA: spatial ICA; CPs: connectivity patterns.

Supplementary Figure 1. Thirty-seven network independent components (displayed on a 3D standard template and shown at the most activated sagittal, coronal and axial slices), subsequently used as the reference template for the spatially constrained ICA analysis (modified from Abrol et al. Neuroimage 2017) [45]. ICA: independent component analysis.

Supplementary Figure 2. Violin-plot distributions for the considered meta-states indexes in the studied groups. The box in the plot represents the interquartile range, the vertical lines the 95% confidence interval whereas the solid black line in the box indicated the median value. For panel A, Y-axis represents the number of meta-states, whereas for panel B, Y-axis represents the number of changepoints. For panel C, Y-axis represents the meta-states span whereas for panel D, Y-axis represents the total cumulative distance traveled (summed L1 distance between successive meta-states) in the state space.

Supplementary Figure 1.

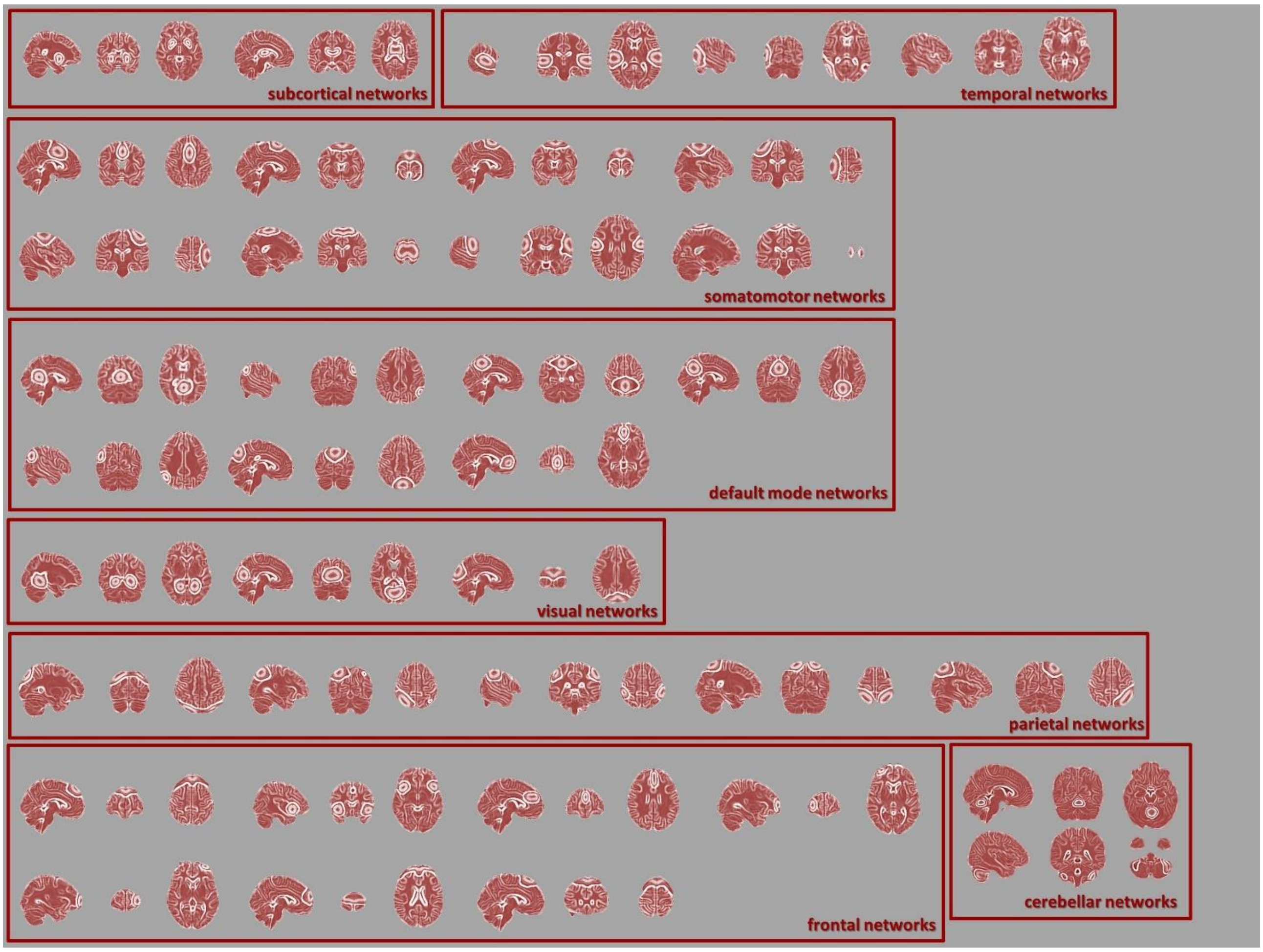

Supplementary Figure 2.

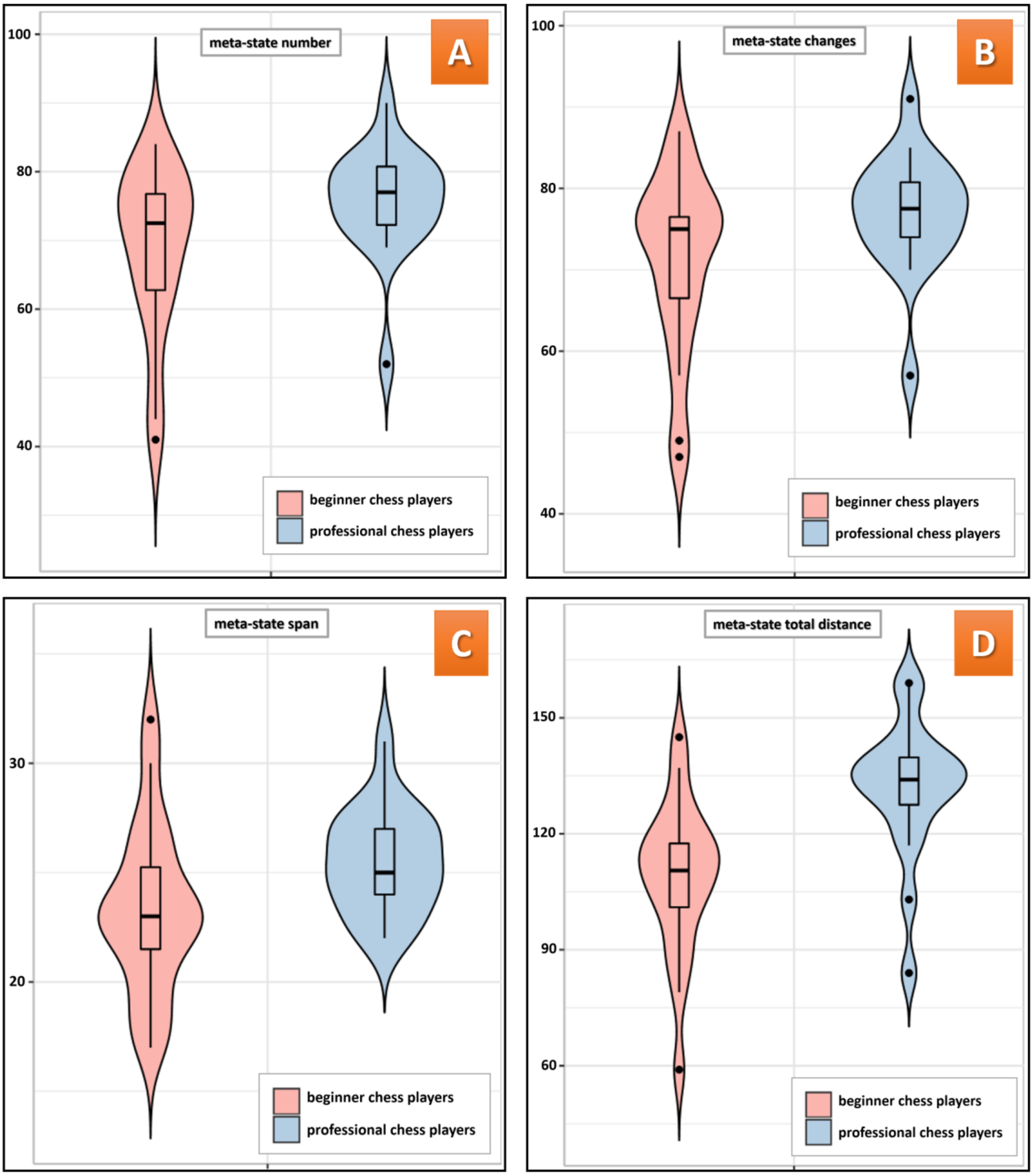

Supplement: Supplementary file 1 — Supplementary Materials. [file 41598_2020_63984_MOESM1_ESM.pdf]
